# Hot Press as a Sustainable Direct Recycling Technique of Aluminium: Mechanical Properties and Surface Integrity

**DOI:** 10.3390/ma10080902

**Published:** 2017-08-03

**Authors:** Nur Kamilah Yusuf, Mohd Amri Lajis, Azlan Ahmad

**Affiliations:** Sustainable Manufacturing and Recycling Technology, Advanced Manufacturing and Materials Center (SMART-AMMC), Universiti Tun Hussein Onn Malaysia (UTHM), Parit Raja 86400, BatuPahat, Johor, Malaysia; nurkamilahyusuf@gmail.com (N.K.Y.); azlan357@gmail.com (A.A.)

**Keywords:** sustainable manufacturing, direct metal recycling, hot press (HP), aluminium AA6061, mechanical properties, surface integrity

## Abstract

Meltless recycling technique has been utilized to overcome the lack of primary resources, focusing on reducing the usage of energy and materials. Hot press was proposed as a novel direct recycling technique which results in astoundingly low energy usage in contrast with conventional recycling. The aim of this study is to prove the technical feasibility of this approach by characterizing the recycled samples. For this purpose, AA6061 aluminium chips were recycled by utilizing hot press process under various operating temperature (T_s_ = 430, 480, and 530 °C) and holding times (t_s_ = 60, 90, and 120 min). The maximum mechanical properties of recycled chip are Ultimate tensile strength (UTS) = 266.78 MPa, Elongation to failure (ETF) = 16.129%, while, for surface integrity of the chips, the calculated microhardness is 81.744 HV, exhibited at T_s_ = 530 °C and t_s_ = 120 min. It is comparable to theoretical AA6061 T4-temper where maximum UTS and microhardness is increased up to 9.27% and 20.48%, respectively. As the desired mechanical properties of forgings can only be obtained by means of a final heat treatment, T5-temper, aging after forging process was employed. Heat treated recycled billet AA6061 (T5-temper) are considered comparable with as-received AA6061 T6, where the value of microhardness (98.649 HV) at 175 °C and 120 min of aging condition was revealed to be greater than 3.18%. Although it is quite early to put a base mainly on the observations in experimental settings, the potential for significant improvement offered by the direct recycling methods for production aluminium scrap can be clearly demonstrated. This overtures perspectives for industrial development of solid state recycling processes as environmentally benign alternatives of current melting based practices.

## 1. Introduction

The carbon dioxide emissions and vast release of solid waste by industrial processes has led to global warming, which brings about adverse effect to human activity. Various industrial processes accounted for approximately 14% of the total carbon dioxide emissions and 20% of the total greenhouse gas emissions in 2010 [1]. According to [2], the assembling division, which lies at the centre of the modern economy, must be made to protect elevated living standards accomplished by industrialized social orders and, in turn, to empower social order to achieve and sustain a similar level of affluence. Hence, the need of decrement in power utilization in mechanical procedures has turned into a central point in the modern world.

Aluminium alloys have been in exigency by the industrial practitioner due to their distinctive properties, which includes good strength and low density compared with steel. Most of them were utilized for food packaging, transportation, food additives, beverage cans, cooking utensils, building medicines, and surgery materials, because of their unique physical and chemical properties [3]. Whenever the demand and application increased, the waste produced from the machining will also significantly increase [4,5,6]. This leads to the production of secondary aluminium to substitute the current use of primary aluminium.

Correspondingly, different methods have emerged in producing secondary aluminium. The most viable aluminium recycling practices in most industries are based on the conventional recycling using a melting technique. It is forecasted that, in 2030, 6.1 megatons of scrap will not be recycled due to high concentration of aluminium alloying elements caused by their inefficient and/or challenging removal during re-melting [7]. The conventional recycling of aluminium is recently less favourable, as the method requires high energy and large number of operations which led to cost increment. Instead of using melting techniques that use a very high temperature to reach the melting point, recycling of wrought aluminium alloys by solid-state is preferable. High energy consumption for conventional aluminium recycling and subsequent refinement has been considered in previous studies. Initially, the solid-state recycling techniques employ the powder metallurgy processes by Gronostajski et al. [8], followed by research from Fogagnolo et al. [9]. Moreover, some of recycling techniques have been studied and show excellent mechanical responses by employing extrusion and powder metallurgy process [10,11,12,13,14,15]. This process chain requires a small amount of energy compared to conventional process chains, using only 5–6 GJ·ton^−1^, which is 5–6% of that needed for the conventional process chain. During the complete conversion of aluminium chips into a compact metal by extrusion, a portion of the chip from which impurities cannot be removed is wasted, amounting to approximately 2%; the extrusion waste can be as high as 3%. Thus, 95% of the aluminium chips are recovered [12]. Spark plasma sintering (SPS) is a new approach of solid-state recycling. The main characteristics of SPS are that the pulsed DC current directly passes through the graphite die and the chip is compacted. The dynamic scrap compaction, combined with electric current-based joule heating, achieved partial fracture of the stable surface oxides, desorption of the entrapped gases and activated the metallic surfaces, resulting in efficient solid-state chip welding eliminating residual porosity [16,17]. On the other hand, a wide research enthusiasm for maintainability is available in the modern technical studies: Life Cycle Assessment (LCA) technique and Design for Environment (DFE) methodology are these days broadly examined in many research laboratories throughout the world [18]. Moreover, Duflou et al. [19] compared the environmental performance of three innovative solid state recycling strategies with the environmental impact of the conventional re-melting approach. The paper demonstrates the noteworthy ecological benefits from utilizing the inventive reusing techniques.

In conjunction with this, hot press showed promising alternative for recycling aluminium as the waste from the machining process [20,21,22]. To validate this, a comparative analysis of an alternative material recycling route hot press process, starting from the same waste materials as the conventional re-melting technique, was studied. The LCA model was created using the Simapro 8.0.5 software for life cycle assessment. The databases contained in the Simapro software provide the LCI data of the raw and process materials used in the background system. The data for conventional technique are collected by the Ecoinvent database combined with published data from the literature. The results of analysis justified that hot press process is evidently gives the significant environmental benefits as compared to the conventional re-melting technique where the Global Warming Potential (GWP) is reduced up to 69.2% [23]. Furthermore, hot press process allowed a good potential of strength and plasticity of aluminium. Recycled aluminium has shown virtuous mechanical and physical properties, when subjected to stern plastic deformation course. Many researchers have agreed that the most significant parameter that must be considered when dealing with aluminium alloys is temperature [14,15,24,25,26]. Theoretically, linear correlation of raise between temperature and mechanical properties of aluminium alloy are ought to occur. Hence, this paper has conducted solid-state direct recycling technique by utilizing hot press process of an aluminium chip with aim to investigate the effect of operating temperature and holding time on the mechanical properties, as well as physical properties of direct recycling of AA6061 aluminium alloy that is potentially to be used as secondary resources.

## 2. Experimental Material and Procedure

### 2.1. Experimental Material and Processing

The chemical composition of commercial aluminium bulk series AA6061-T6 is depicted in Table 1. It was measured from energy-dispersive X-ray spectroscopy (EDS) using Hitachi SU8000 (Hitachi, Tokyo, Japan), and is the average of three EDS spots. Copper intermetallic compounds in low concentration detected in all EDS spots. Parts of copper, silicon, magnesium, manganese, and iron intermetallic have been detected. Aluminium exhibited as a main element (94.9–95.4 wt %). In comparison to the compounds of detected elements with ASM handbook material data (2006), this verified that the material used in this research is AA6061 aluminium alloy.

**Figure 1:**
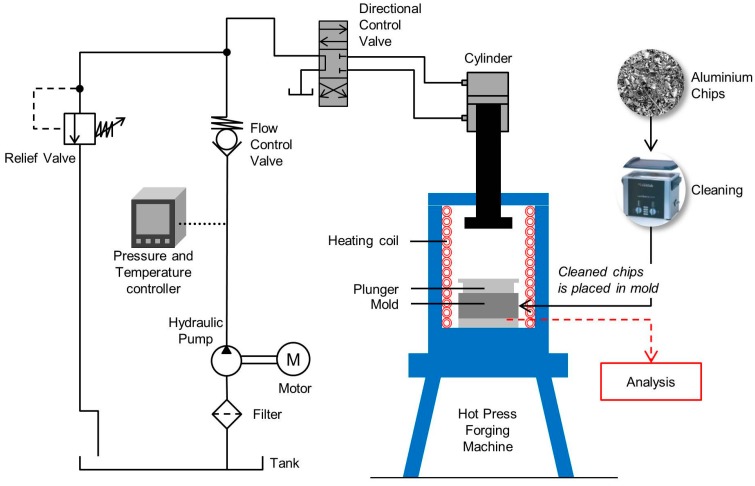
Direct recycling hot press process.

The AA6061 bulk material was milled to produce medium size aluminium chips with average sizes 5.20 mm × 1.097 mm × 0.091 mm in Figure 1. The milling took place in the Sodick-MC430L high speed machining, with the cutting speed, v, of 110 m/min; feed rate, f, of 0.05 mm/tooth; and the depth of cut of 1.0 mm. The aluminium chips produced by machining should not contain any impurities and dirt because these would alter their chemical composition and subsequently impair the diffusion bonding of the chips during solid-state recycling. A cleaning process in acetone (C_3_H_6_O) begins as soon the chip leaves the milling machine, following ASTM G131-96. The drying process is 30 min in thermal drying oven at 60 °C. The cleaned aluminium chip was poured into the mould and the plunge is fixed accordingly.

**Figure 2:**
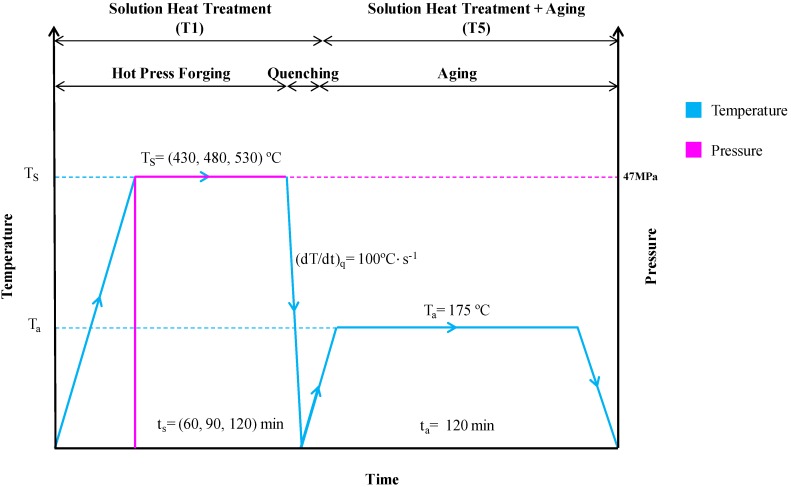
Direct recycling hot press diagram.

Hot press process (Figure 1) was executed with the steady pressure at 47 MPa (around 35 tons) and four times pre-compacting cycle. Indeed, Figure 2 shows the process diagram of direct recycling hot press. The temperatures selected are 430, 480, and 530 °C after considering the optimum operating temperature for hot forging and heat treatment process. In addition, the three selected holding times are 60, 90 and 120 min. Immediately after the forging process (solution heat treatment), the specimen will be quenched in water at quench rate 100 °C/s to perform rapid cooling into room temperature followed by artificial aging at temperature 175 °C with 120-min duration. 

Samples with the corresponding designated different parameter setting are represented in Table 2. The recycled specimen after hot forging process is denoted as T1-temper at which the aluminium were cooled from an elevated-temperature shaping process and naturally aged to a substantially stable condition [27]. These recycled specimens are considered comparable with theoretical AA6061-T4 temper in terms of aluminium being the solution heat treated and naturally aged to a substantially stable condition [27] to observe the potential of recycled material to be used as secondary resources. The complete cycle of heat treated recycled specimen is denoted as T5-temper, in which the aluminium was cooled from an elevated temperature shaping process and subsequently artificially aged [27]. This heat treated recycled billet are considered comparable with as-received AA6061-T6 and denoted as AR-T6. Analyses include tensile test, ultimate tensile strength, and elongation to failure for mechanical properties, while microhardness and density are considered for surface integrity analyses.

### 2.2. Material Characterization Measurement

#### 2.2.1. Mechanical Characterization

**Figure 3:**
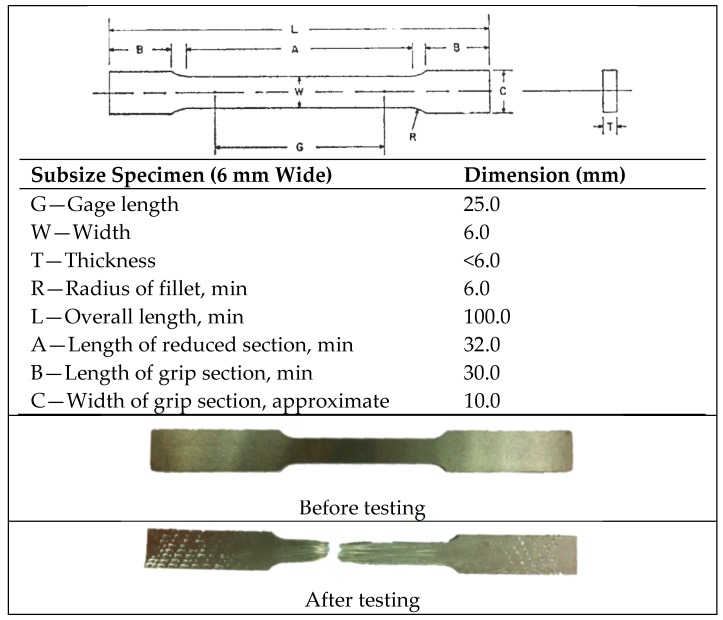
Tensile specimen dimension after recycled process.

The forming procedure for arrangement for tensile testing in this paper abide by the following standard of Standard Test Methods for Tension Testing of Metallic Material (ASTM E8M, 2012), and the dimensions are shown in Figure 3. Tensile tests were performed using universal testing machine (UN-7001-LS, GOTECH Testing Machines Inc., Taichung, Taiwan) using a 25 kN load cell with gauge length 25 mm was used to record the strain. Tensile tests were performed at speeds 5 mm/min.

#### 2.2.2. Microstructural Characterization

The microstructure analysis followed the Standard Guide for Preparation of Metallographic Specimens (American Society for Testing and Materials E3, 2001) and Standard Test Method for Macroetching Metals and Alloys (American Society for Testing and Materials E340, 2006). Light optical microscope (Nikon Eclipse LV150 NL, Nikon Metrology NV, Tokyo, Japan) was used to quantify the microstructural of the recycled samples. The samples were prepared for metallographic observations by grinding the sample consecutively with 60, 240, 400, 600 and 1200 SiC papers, and then polishing with 6 and 1 µm DIAMAT Polycrystalline Diamond cloths. Then, 0.02 µm SIAMAT Noncrystalline Colloidal Silica was used for finishing. The samples were etched with Barker’s reagent to show the grain boundary developed by dynamic recovery or recrystallization.

#### 2.2.3. Hardness

The Vickers hardness test was selected to measure the microhardness of recycled AA6061 aluminium alloy in this study. It was conducted by pressing an indenter into the surface of the tested material using a controlled force, followed by removal of the indenter, and finally the measurement of the indentation size. The analysis was done based on the Standard Test Method for Knoop and Vickers Hardness of Materials (American Society for Testing and Materials E384, 2011). Hardness test samples were cut into 10.0 mm × 10.0 mm × 15.0 mm using an abrasive cutter under the flow of cooling liquid to prevent frictional heating. Vickers microhardness measurements was made with a 2.943 N load in the span of 10 seconds holding time were used to monitor the hardness and data reported represent an average of at least 10 measurements.

#### 2.2.4. Density

The experimental density of the composites was obtained using Archimedean method of weighing small pieces cut from the composite, first in air and then in water. The density of the sample is measured using density balance (HR-250AZ) from, and the actual mass and apparent weight of specimen are calculated while immersed in water.

## 3. Results and Discussion

### 3.1. Mechanical Properties

**Figure 4:**
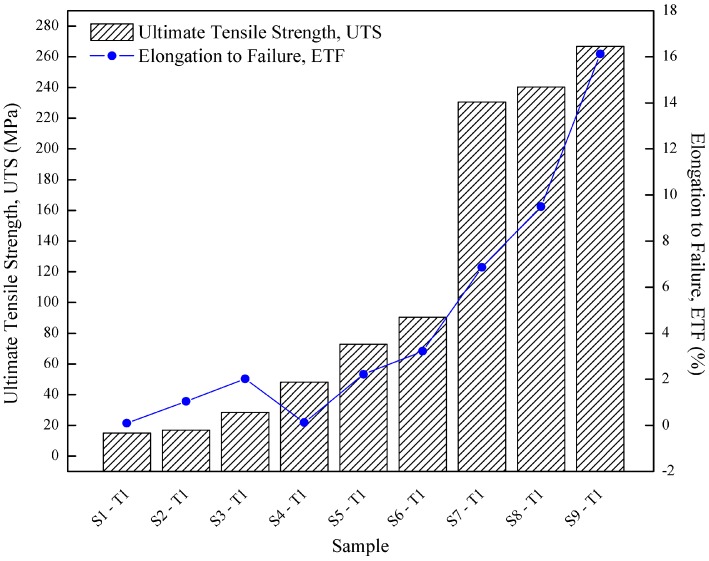
Tensile results of recycled specimen at different operating time and temperature.

Table 3 shows the mechanical properties, including ultimate tensile strength (UTS), elongation to failure (ETF) and microhardness as a function of artificial aging time for recycled specimen. Figure 4 shows the result for UTS and ETF for different operating temperature and holding time. The results in Figure 4 show that the tensile strength is very sensitive to temperature, with an increasing trend when the temperature increases from 430 to 530 °C where UTS increased with the increment of temperature. The inclination trend is depicted as holding time at its maximum (120 min) where at S3-T1 (28.44 MPa) surged up to 266.78 MPa at S9-T1. At holding time of 60 min, the value of UTS increased with increasing operating temperature ranging from S1-T1 (14.97 MPa) to S9-T1 (230.55 MPa). The ETF has a linear trend with increment of temperature, which is similar to the UTS trend. As can be seen, at the maximum holding time (120 min), ETF for S3-T1 (2.027%) elevated up to S9-T1 (16.129%) at 530 °C. At holding time 60 min, the value of ETF increased with the increasing operating temperature, exhibiting 6.769% from S1-T1 (0.091%) to S7-T1 (6.860%). 

As depicted in Figure 4, the results on the effect of operating temperature clearly give the significant impact to the forging process by proving the fact that at constant holding times, value of UTS and surged up to 215.58–238.34 MPa and ETF 6.769–14.102% by increasing operating temperature from 430 °C to 530 °C. The primary cause of the increase in strength is the operating temperature exceeded the solvus temperature: this causes the precipitates to re-dissolve and results in solute solution hardening. The operating forging temperatures in this study were selected between the solidus and the recrystallization temperature. The process requires high temperature, however it should not be higher than liquid temperature as well as be given sufficient time for phases containing copper and/or magnesium to dissolve completely so that a nearly homogenous solid solution is achieved. The temperature and time must be well controlled, because either insufficiency or excessiveness may cause coarsening and decreasing precipitate [28]. Solubility increases with increase in temperature, so conducting in lower temperatures will give worse properties [29]. Furthermore, at high temperature, solid solution strengthening is expected to have a major effect. Friis et al. [30] and Langkruis et al. [31] stated that the tensile tests represent the occurrence of significant hardening, caused by severe plastic strain induced in the samples, in the deformed state. Rometsch and Schaffer [32] had analysed the yield strength with an assumption of independent contributions of solid solution hardening, precipitation hardening, and intrinsic strength. Meng [33] also agreed that the increment of UTS were affected by the up rise of heating temperature by stated that during solutionizing, solute atoms were dissolved in the Aluminium matrix and retained in a metastable state. These fine clusters (precipitates) act as a barrier for dislocation movement, causing an enhancement of initial strength. Moreover, Oppenheim et al. [34] agreed that, below 470 °C, lowest strength and hardness were produced by indicating that a significant amount of the solute did not go in solid solution at this low temperature, whereas higher strength and hardness were exhibited at high temperature up to 500 °C.

The same trend is observed for the holding time effects in comparison to UTS at which it demonstrated the effect of holding time, as shown in Figure 4. At the operating temperature 430 °C, the value of UTS marginally raised (13.47 MPa) with the increase of holding time from S1-T1 (14.97 MPa) to S3-T1 (28.44 MPa). At the maximum operating temperature of 530 °C, the value of UTS slightly increased from 230.55 MPa at S7-T1 to 266.78 MPa at S9-T1. Meanwhile, a similar ascending trend for ETF demonstrated the effect of holding time. At the operating temperature 430°C, the value of ETF slightly raised (1.936%) with the risen of holding time from S1-T1 (0.091%) to S3-T1 (2.027%). At the maximum operating temperature of 530 °C, the value of ETF increased from 6.860% in S7-T1 to 16.129% in S9-T1. On the other hand, when comparing the minimum–maximum parameter (S1-T1 with S9-T1), the value of UTS is observed to increase dramatically from 14.97 MPa to 266.78 MPa. 

**Figure 5:**
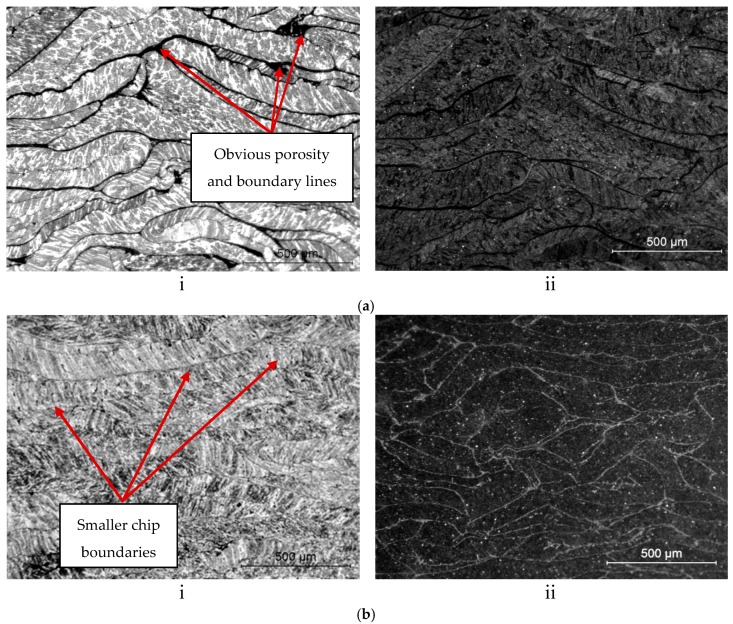
Microstructure of minimum–maximum parameter: (**i**) bright field; and (**ii**) under polarized light. (**a**) S1-T1 (430 °C/60 min); (**b**) S9-T1 (530 °C/120 min).

Figure 5 shows microstructure at S1-T1 (430 °C/60 min) and S9-T1 (530 °C/120 min). At S9-T1 the average grain diameter is 30.78 µm, whereas, at S1-T1, the grain diameter revealed to be bigger (61.70 µm). At a minimum parameter, the porosity between the chips are greatly observable, as shown in Figure 5a. This shows that, at low temperature, chip is simply interlocking instead of consolidating. On the contrary, the recycled aluminium profiles at different operating temperature show that the high temperature leads to recrystallized grain boundary where porosity between the chips is less apparent at S9-T1 in Figure 5b. The size of the grain boundary became closer between the chips, which shows that, at maximum parameter, the solid-state welding of the chip is enhanced. The orientation of the grain boundary is closer between the chips, which proved that, at maximum temperature of 530 °C, the solid-state welding of the chip is enhanced. Russel and Kok [35] have compared the stress-strain behaviour of single crystal and polycrystalline aluminium tensile specimens for different grain size. In general, small grain size produced close space barrier to dislocation and their study conclude that the closer the spaced barrier to dislocations, the greater strength will be produced. However, it is known that dynamic recrystallization occurs during hot extrusion for Al-Mg alloys. Thus, it is acceptable that the higher strength produced at 530 °C recycled specimen is attributed to grain refinement strengthening and particle-dispersion strengthening [36]. Thus, the fine-grained microstructures of recycled specimens from dynamic recrystallization during hot working will eventually lead to higher UTS.

During solutionizing at temperature of 530 °C, the cold-deformed specimens led to recrystallization. It was known that the higher the strains, the lower the recrystallized grain size [37]. The results obtained during tensile tests shows that the structure affects mechanical properties. Coarse structure led to the worst tensile strength in comparison with the finer microstructures. This is proven by UTS exhibiting only 14.97 MPa at minimum parameter (S1-T1). However, the grain size is shown to be the biggest at 61.70 µm. As mentioned before, the operating forging temperatures for hot working were selected to comprise the temperature between solidus and recrystallization. In the case of AA6061 aluminium alloy physical properties designation, recrystallization temperature started from 450 °C and solidus region started at 580 °C [27]. This is because during recrystallization temperature, the microstructure condition started to enter the second phase of precipitation. Precipitation is achieved by heating the alloy below the solidus line at a suitable temperature and time. In this process, solute atoms diffuse to form small precipitates [38]. These fine precipitates act as a barrier for dislocation movement, causing an enhancement of initial strength [33].

**Figure 6:**
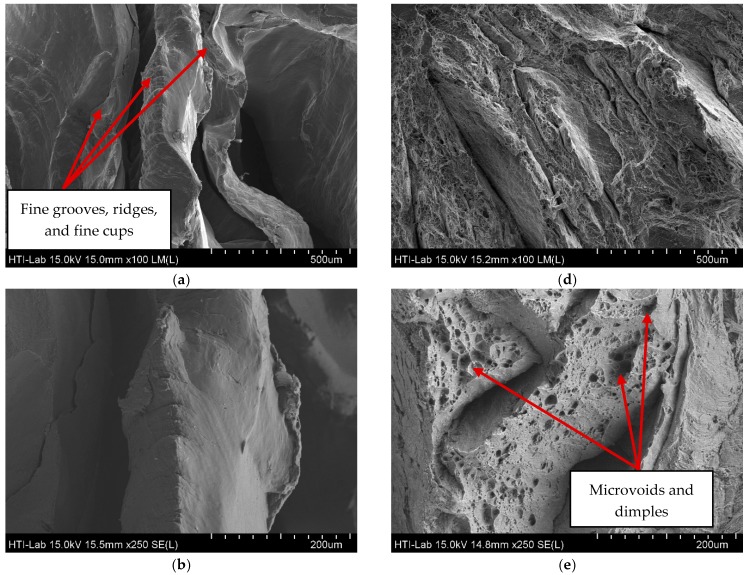

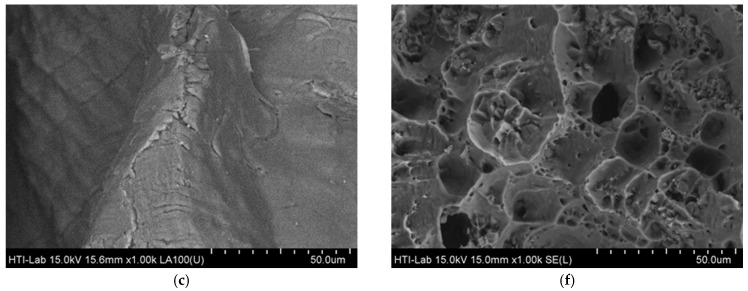
FESEM micrographs showing the fracture surface of minimum–maximum parameter S1-T1 (430 °C/60 min) and S9-T1 (530 °C/120 min): (**a**) S1-T1 (430 °C/60 min); (**b**) S1-T1 (430 °C/60 min); (**c**) S1-T1 (430 °C/60 min); (**d**) S9-T1 (530 °C/120 min); (**e**) S9-T1 (530 °C/120 min); and (**f**) S9-T1 (530 °C/120 min).

As illustrated in Figure 4, when comparing the minimum–maximum parameter (S1-T1 and S9-T1), the value of ETF increased dramatically from 0.091% to 16.129%. Figure 6 shows the FESEM image of the fractured surfaces of the minimum (SI-T1) and maximum (S9-T1) parameter setting. Magnification of 100× is an overview of the fracture surface, 250× is the periphery coarse topology of the fracture surface and 1000× is equiaxed dimples.

At low operating temperatures and holding times (S1-T1), the fractured surfaces obviously reveal as flat and shallow, and there are no dimples at all. This type of tear fracture suggested that the deformation occurs in bands through the grains, which is expected when the dislocation governed the deformation by cutting through the strengthening particles. It is also observed that a little gross of plastic deformation and the fracture takes place along the crystallographic plane (cleavage plane) in which the normal stress is maximized. It appears that no gross plastic deformation and fracture takes place along the crystallographic plane (cleavage plane) on which the normal stress is maximized. At low magnification, these flat areas exhibit fine grooves, ridges and fine cups, as shown in Figure 6a. This supports the results of ETF at which at S1-T1, the result is as low as 0.09%. This type of fracture is normally classified as brittle deformation [13]. As observed at maximum parameter (S9-T1), it consists of many microvoids and dimples indicating the ductile fracture mode. The presence of large and deep dimples in the fractured surface in Figure 6e is evidence for large fluent strain of the specimen (ETF 16.13%). However, the dimples did not form uniformly in this condition and the dimples of different size are apparent in this figure.

### 3.2. Surface Integrity

**Figure 7:**
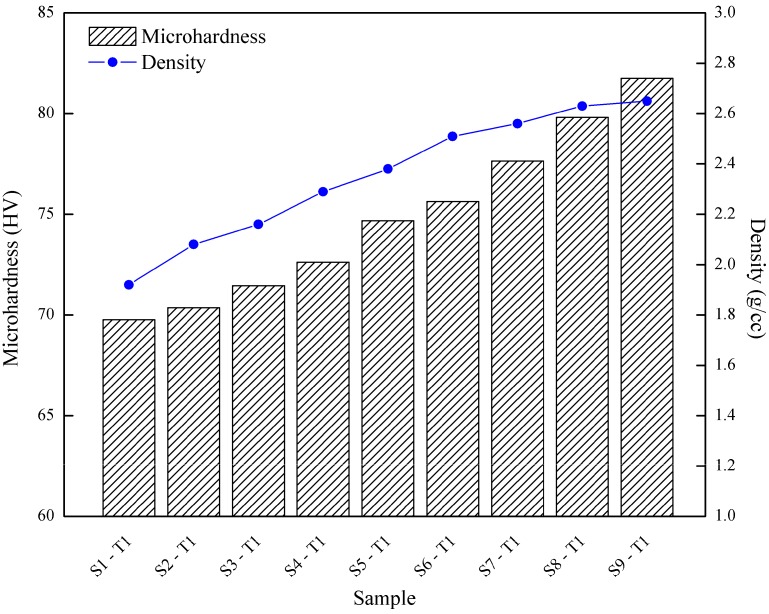
Results of microhardness and density at different operating times and temperatures.

Figure 7 shows the overall results for the effects of operating temperature and holding time on the surface integrity. Surface integrity is the sum of all the elements that describe all the conditions existing on or at the surface of the material resulting from controlled manufacturing process. The surface integrity of material greatly influences mechanical properties of alloys. From the results, it shows that the value of microhardness increased with the increment of temperature. At holding time 60 min, the value of microhardness increased with increasing operating temperature, yielding from S1-T1 (69.761 HV) to S7-T1 (77.642 HV). The same trend is observed as the holding time at maximum of 120 min: S3-T1 (71.455 HV) surged up to 81.744 HV at S9-T1. The effect of parameter obviously gives the notable impact to the forging process when the increment in microhardness raised up to 11.983 HV. From the previous study, the relationship between hardness and strength was studied by several researchers [13], which was confirmed by the tensile test results where similar trends were observed in hardness and UTS. Tan and Ogel [37] stated that, as temperature increases, more solid dissolves in the matrix for super saturation, due to increased diffusion kinetics and higher solubility limits at higher temperatures.

Meanwhile, as illustrated in Figure 7, a similar upward trend for microhardness demonstrated the effect of holding time. At the operating temperature 430 °C, the value of microhardness marginally raised with the increased of holding time of 60 min at S1-T1 (69.761 HV) to 120 min at S3-T1 (71.455 HV). At the maximum operating temperature 530 °C, the value of microhardness slightly increased from at S7-T1 (77.642 HV) to 81.744 HV at S9-T1. Below 530 °C, there was a tendency towards increasing hardness with increasing the holding time, however the trend was opposite for temperatures above and this was agreed by several researchers [28,29,39]. Furthermore, when comparing the minimum–maximum parameter (S1-T1 and S9-T1) the value of microhardness increased from 69.761 HV to 81.744 HV. The elastic properties of specimens were slightly higher compared to the expected and measured values of the theoretical value, as a result of the precipitation and oxide dispersion hardening. The material is stiffer due to the high oxide content which is dispersed within the material [16].

As observed for density analysis in Figure 7, at holding time of 120 min, the value of density increased with the increasing of operating temperature, showing the increment from S3-T1 (2.16 g/cc) elevated to 2.65 g/cc at S9-T1. At the holding time of 60 min, density raised up from S1-T1 (1.93 g/cc) to S7-T1 (2.56 g/cc). Meanwhile, a similar ascending trend for density is demonstrated as the effect of holding time. At operating temperature of 430 °C, the value of density increased with increasing holding time at S1-T1 (1.92 g/cc) to S3-T1 (2.16 g/cc). At the maximum operating temperature 530 °C, the value of density slightly increased about from 2.56 g/cc at S7-T1 to 2.65 g/cc at S9-T1. It is obviously shown that the density increase whenever the holding time and the temperature increase. Insufficient temperature and holding time will eventually result in the alloy having large voids, which leads to low density [40]. This retention process is assisted by raising the holding time, allowing the alloy to have better bonding between the chips. Great combination of high temperature and high holding time permits the composite to exert high density.

When comparing the minimum–maximum parameter (S1-T1 and S9-T1), as shown in Figure 7, the value of density increased from 1.92 g/cc to 2.65 g/cc. This can be referred to the microstructure analysis; the obvious porosity in Figure 5a gives the lower density value at minimum parameter, while closer grain boundary structure in Figure 5b at maximum parameter produced high density. Unfortunately, the value of maximum density only exhibited 2.65 g/cc at maximum parameter (S9-T1) which it was below the theoretical value; 2.7 g/cc (AA6061 T4—from ASM, 2009). This phenomena agreed well with the study from Ceschini et al. [41], which showed a slight reduction of the porosity, after forging, without evidence of material damage induced by the plastic deformation, both in terms of reinforcement/matrix de-cohesion or particle cracking. Moreover, the high oxide content of the chips can explain this. Fine-form scrap has a very high surface area-to-volume ratio, and, thus, the oxide content is much higher than for solid samples [16].

**Figure 8:**
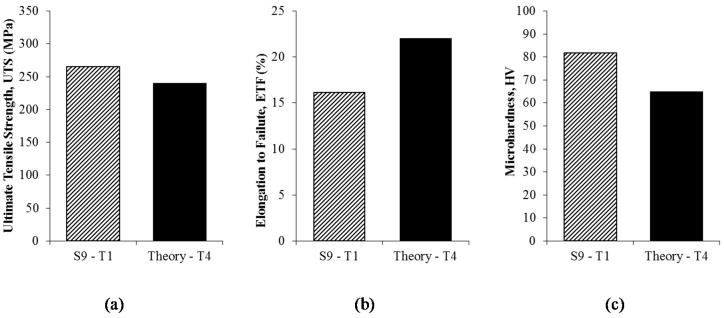
Results of recycled aluminium and theoretical value and as-received AA6061: (**a**) ultimate tensile strength; (**b**) elongation to failure; and (**c**) microhardness.

In addition, the maximum UTS peaked at 266.78 MPa (S9-T1), which shows an increment of 9.27% from the theoretical value (240 MPa (Theory T4)), as shown in Figure 8. Unfortunately, the value of maximum ETF only exhibited 16.129% at maximum parameter (S9-T1) which was 22% below the theoretical value (Theory T4). The value of maximum microhardness reaches 81.744 HV (S9-T1), which is 20.48% above of the theoretical (65 HV) (Theory T4). The increment of UTS and microhardness value clarify the potential ability of hot press process to give a notable potential to be utilized as one of the recycling method of aluminium.

### 3.3. Post-Process (Artificial Aging-T5 Temper)

**Figure 9:**
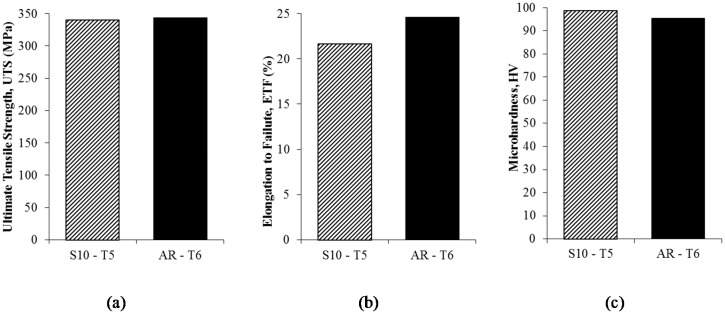
Results of heat treated recycled aluminium and as-received AA6061: (**a**) ultimate tensile strength; (**b**) elongation to failure; and (**c**) microhardness.

The desired mechanical properties of forgings can be obtained only by means of final heat treatment. By performing heat treatment to the recycled sample immediately after hot press process S10-T5, it is interesting to note that the strength behaviour changes dramatically compared to the heat-treated sample, reaching the strength of 340.47 MPa and the ETF increased to 21.70%, as shown in Figure 9. This value is comparable to as-received AR-T6. The maximum hardness exhibited 98.649 HV for heat-treated sample S10-T5 at 120 min aging time and 175 °C aging temperature. An increase in hardness and strength could be explained by a diffusion assisted mechanism, and by the hindrance of dislocation by impurity atoms, i.e., foreign particle of the second phase, since the material after quenching from 530 °C (solution heat treatment) will have excessive vacancy concentration [42]. Rafiq et al. [43] proved that the increase of aging time and temperature will lead to the increasing of GP Zone density. Hence, the degree of irregularity in the lattices will cause an increase in the mechanical properties of the aluminium alloy. Compared to the maximum value of microhardness at S10-T5, the value is 3.18% greater to as-received AR-T6 (95.512 HV).

**Figure 10:**
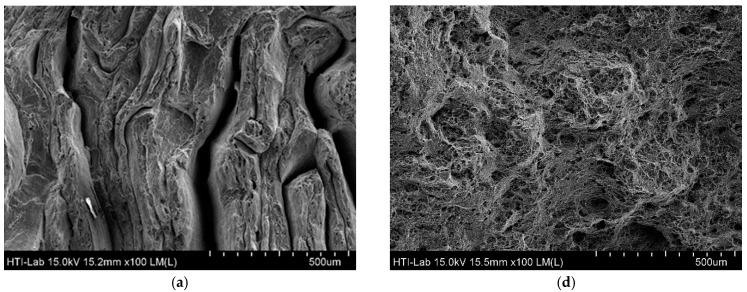

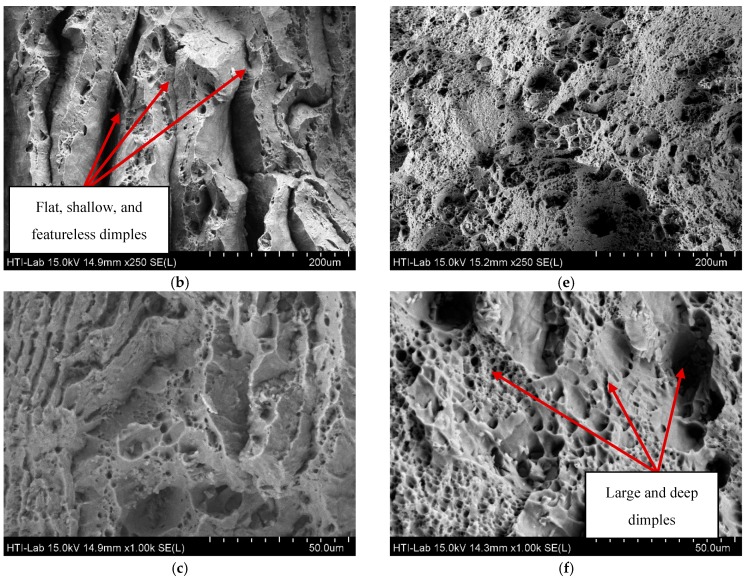
FESEM micrographs showing the fracture surface of the profiles at the heat-treated sample S10-T5 and as-received AR-T6: (**a**) S10-T5 (heat treated)−100×; (**b**) S10-T5 (heat treated)-250×; (**c**) S10-T5 (heat treated)-1000×; (**d**) AR-T6 (As-received)-100×; (**e**) AR-T6 (As-received)-250×; and (**f**) AR-T6 (As-received)-1000×.

Consequently, Figure 10 shows the FESEM images of the fractured surfaces for S10-T5 at 120 min of aging and as-received AR-T6. From S10-T5, fractured surfaces reveal as flat, shallow, and featureless dimples. This type of tear fracture suggests that the deformation occurs in bands through the grains, which is expected as dislocation governs the deformation, cutting through the strengthening particles [44]. At high magnification, these flat areas exhibit fine grooves, ridges and fine cups, as shown in Figure 10b. This type of fracture is normally classified as brittle deformation [13]. This parallels the results for ETF S10-T5, which was 22.39% lower than the as-received AR-T6. In as-received AR-T6 images, numerous microvoids and dimples can be observed, indicating the ductile fracture mode. The presence of large and deep dimples in the fractured surface in Figure 10f proved the large fluent strain of the specimen, which is 24.62%. However, the dimples are not formed uniformly in this condition and the dimples of different sizes are apparent in this figure.

**Figure 11:**
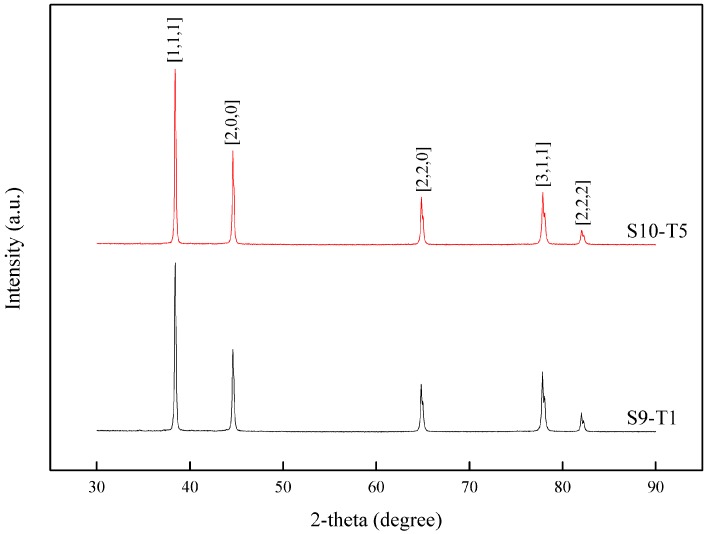
XRD spectra of recycled specimen for S9-T1 and S10-T5.

The EDX analysis of the white regions on the fractured surfaces in Table 1 revealed the presence of elements Fe, Mn and Cu in the basis phase. As anticipated, phases containing significant quantities of heavy elements appeared to be brighter than phases containing light elements (lower atomic number elements) [45]. To detect the constituting phases made up of these elements, X-ray diffraction (XRD) analysis was performed for both solutionised S9-T1 and heat treated recycled billet S10-T5 (Figure 11). X-ray diffraction (XRD) technique can be used to characterize the microstructural evolutions in terms of studying the maximum peak intensity/changes. Based on composition, the predominant equilibrium second phase in AA6061 are shown to be Al, AlFeSi and AlFeMn, as depicted in the Appendix. Comparing the intensity of the detected phases for S9-T1 and S10-T5 heat treated sample, intensity of the (111) and (200) pole increased, as shown in Figure 11. Shokuhfar et al. [45], Kuijpers et al. [46], and B. Lin et al. [47] define (200) pole as β-AlFeSi, which is also known as a hard phase. This analysis demonstrated the relative number of phases in the alloy confirms that the β-AlFeSi phase increased by heat treatment at S10-T5. The solutionised sample S9-T1 shows that the precipitates are not completely dissolved. It is known that grain and sub-grain boundary migration has an important role in the development of the (200) texture [48]. The soft phase has less resistance to the plastic flow than the hard phase. Moreover, the load redistribution is taken up by the hard phase which also weakens the ductility and workability of 6xxx alloys. In addition to AlFeSi phase, in 6xxx aluminium alloys, Mn is interchangeable with Fe atoms to form the α-Al(FeMn)Si phase. As the contrast between these two phases is very low, distinguishing the phase type is very difficult by automated systems [49]. Shokuhfar et al. [45] and Ezatpour et al. [48] agreed with these results, demonstrating the relative amount of phases in the alloy confirms that the hard β-AlFeSi phase increased by heat treatment. This observation confirms both increasing strength and microhardness after heat treatment. Therefore, the increased strength and microhardness, comparing S9-T1 and heat treated S10-T5 condition, are due to the increase in the hard phase.

## 4. Conclusions

Conclusively, direct recycled AA6061-T6 chip by utilizing hot press process shows great effects on the mechanical properties and surface integrity. The conclusions can be summarized as follow:

1. Results on the effect of operating temperature give most significant impact to the forging process:
  - UTS increased 89.51–93.35% from 14.97–266.78 MPa by increasing the operating temperature from 430 °C to 530 °C.
  - ETF increased 86.32–98.67% from 0.091–16.12% by increasing the operating temperature from 430 °C to 530 °C.
  - Microhardness reached 7.88–10.28 HV by increasing the operating temperature from 430 °C to 530 °C.
2. Maximum mechanical properties and surface integrity of recycled chip (T1-temper) are considered comparable with theoretical AA6061 T4-temper:
  - Maximum UTS are notably raised up to 9.27% (266.78 MPa) at parameter condition (530 °C/120 min).
  - Maximum hardness surged up to 20.48% (81.74 HV) at parameter condition (530 °C/120 min).
3. Heat treated recycled billet (T5-temper) are considered comparable with as-received AA6061 T6-temper:
  - Value of microhardness peak after 175 °C for 120 min aging increased 3.18% (98.64 HV).
  - Value of UTS (340.47 MPa) and ETF (21.70%) reached close to the as-received value.

Although this is only the foundation of experimental observations, its potential as an alternative technique to the direct recycling methods for producing scrap can be clearly demonstrated. This analysis justified that hot press process evidently has significant benefits compared to conventional recycling. Improved energy efficiency is currently the focus of on-going research, which allows further impact reductions. This study offers perspectives for industrial development of solid state recycling processes as environmentally benign alternatives for the current melting based practices.

## Figures and Tables

**Table 1:** Average chemical element of as-received AA6061-T6 matrix

Elements	Weight Percent (wt %)
Aluminium	95.03
Magnesium	1.17
Silicon	0.34
Iron	0.46
Copper	0.15
Manganese	0.14
Others	Balance

**Table 2:** Sample designation for different parameter setting.

Sample Designation	Solution Heat Treatment (Hot Press)		Solution Heat Treatment + Aging	
	Operating Temperature, T_s_ (°C)	Holding Time, t_s_ (min)	Aging Temp, T_a_ (°C)	Aging Time, t_a_ (min)
S1-T1	430	60	-	-
S2-T1	430	90	-	-
S3-T1	430	120	-	-
S4-T1	480	60	-	-
S5-T1	480	90	-	-
S6-T1	480	120	-	-
S7-T1	530	60	-	-
S8-T1	530	90	-	-
S9-T1	530	120	-	-
S10-T5	530	120	175	120
Theory-T4	ASM Theory AA6061-T4			
AR-T6	As-Received AA6061-T6			

**Table 3:** Results for tensile properties for all samples.

Sample Designation	Ultimate Tensile Strength, UTS	Elongation to Failure, ETF	Microhardness	Density
	(MPa)	(%)	HV	g/cc
S1-T1	14.97	0.09	69.76	1.92
S2-T1	16.91	1.04	70.37	2.08
S3-T1	28.44	2.02	71.45	2.16
S4-T1	48.11	0.12	72.62	2.29
S5-T1	72.84	2.22	74.69	2.38
S6-T1	90.42	3.22	75.63	2.51
S7-T1	230.55	6.86	77.64	2.56
S8-T1	240.27	9.50	79.81	2.63
S9-T1	266.78	16.12	81.74	2.65
S10-T5	340.47	21.70	98.64	2.69
Theory-T4	240.00	22.00	65.00	2.70
AR-T6	343.43	24.62	95.51	2.67
